# Are the Terms Major and Minor Allergens Useful for Precision Allergology?

**DOI:** 10.3389/fimmu.2021.651500

**Published:** 2021-03-08

**Authors:** Luis Caraballo, Rudolf Valenta, Nathalie Acevedo, Josefina Zakzuk

**Affiliations:** ^1^Institute for Immunological Research, University of Cartagena, Cartagena, Colombia; ^2^Department of Pathophysiology and Allergy Research, Division of Immunopathology, Center for Pathophysiology, Infectiology and Immunology, Medical University of Vienna, Vienna, Austria; ^3^Laboratory for Immunopathology, Department of Clinical Immunology and Allergology, Sechenov First Moscow State Medical University, Moscow, Russia; ^4^Karl Landsteiner University of Health Sciences, Krems, Austria; ^5^NRC Institute of Immunology FMBA of Russia, Moscow, Russia

**Keywords:** allergen, major and minor, immunoglobulin E, asthma, specific immunoglobulin E, precision medicine, allergenic activity, allergenicity

## Introduction

Scientific advances in the molecular characterization of IgE-binding molecules, together with the development of precision medicine, have revealed great limitations on classifying allergens as majors and minors, which considers mainly their IgE-binding frequency. This has become more evident with the discovery of strong allergenic activity in molecules with low IgE-binding frequency, which activate non-IgE-mediated inflammatory mechanisms. Therefore, it is necessary to make the pertinent modifications and to outline a different way to interpret the IgE-binding property of allergens, according to precision allergology. This article analyzes the origin and evolution of the current classification, supports the need for a different way to evaluate the clinical importance of allergens and suggests abandoning the practice of classifying them based on their IgE binding frequency. Since house dust mites (HDM) are so important as inducers of asthma and allergic rhinitis, the analysis is based on the IgE-binding components from *Dermatophagoides pteronyssinus* and *Blomia tropicalis*. However, the main concepts and the conclusions are valid for most allergens from other sources, even taking into account their peculiarities.

## The Current Major and Minor Allergen Classification

Nowadays, one of the main aspects of the characterization of an allergen is defining its IgE-binding frequency and classifying it as major (>50% IgE-binding) or minor (<50% IgE-binding), as suggested by H. Lowenstein in 1978 (1). In a review about the clinical impact of HDM allergens, W. Thomas classified them as serodominant, mid-tier, minor, and allergens of unknown importance (2). These classifications are not official declarations of any scientific society or the WHO/IUIS Allergen Nomenclature Committee. In fact, in a recent publication from this Committee, the authors make the following statement: “*it is important to note that as a committee on nomenclature, the mission is to provide a framework for consistent allergen identification and not to pass judgement on whether an allergen is a major, mid-tier, or minor allergen. The scientific discussion about the merits of a particular allergen is simply facilitated by accurate nomenclature to identify the molecule in question”* (3).

## Origins and Evolution of the Current Classification

In an updating chapter on the main characteristics of allergens, Professor D. Marsh, trying to clarify the *in vogue* idea of “major allergen,” defined it as an allergen that induced immediate skin test responses in >90% of allergic individuals in contrast to a “minor” allergen, to which <20% of patients gave skin test responses (4). Since his chapter, the classification has been changed several times (5); mainly in relation to the limiting percentages of IgE reactivity for major allergen (1, 2). Other interpretations have been made, some of them, as analyzed by Prof. L. Berrens, lacking scientific clarity and plenty of arbitrariness (5), which led him making the following reflection: “*One is left with the impression that a major allergen is something that a particular investigator wishes it to be, within the constraints of the clinical, epidemiological, technological or economical parameters available to him. I have this singular feeling that maybe there is no such thing as a major allergen.”*

An important point is that all modifications maintained the allergen, not the patient, as the center of attention, just like if being major were not an epidemiological parameter influenced by the wide heterogeneity of the IgE responses. In the original definition, the IgE reactivity was detected by skin test, which meant more weight to infer clinical significance. Over time the IgE-binding detection has been switched to *in vitro* assays, without any change in the original perception of the relationship between IgE reactivity (as detected by skin test) and clinical impact. Then we are using a classification that, from the beginning, has had limitations that have increased in parallel with the advances in allergology. The need for changing this classification has been claimed by several investigators, mostly in the field of HDM-induced allergy (2, 6–8).

## Are the Terms Major and Minor Allergens Useful?

The IgE reactivity has been associated with the clinical importance of allergens because of the great role of adaptive immunity in the allergic response that was, at the beginnings, mainly related to the humoral (IgE) component of the Th2 response. Hence it has been assumed that specific IgE is like a proxy embodying almost all the properties and circumstances that make a molecule allergenic (Table 1). In fact, IgE reactivity is, by definition, tightly linked to an allergen (its allergenicity), no matter if this reactivity has clinical impact. Allergenicity is determined by the genetic background (genotype) of the host and as occurs with atopy, is not necessary associated to allergic disease.

**Table 1:** Factors influencing the HDM induction of a Th2 response.

1. Allergen concentration in the natural source a. House dust mite genotype b. Microenvironment in which mite grows c. Diet and mite metabolism d. Climate and stress conditions of mites e. Predominant allergen isoforms 2. Allergen concentration in the environment a. Molecule stability b. Molecule volatility 3. Persistence and level of exposure a. Life cycle of mite b. Climate and geographic location c. Type of housing and personal habits 4. Biological activity of the allergen a. Enzymes b. Adjuvants of Th2 response c. Suppressors of Th2 response d. Other environmental factors present in the household dust 5. Patient's genomic and epigenetic machinery a. Predisposition to IgE biased response b. Predisposition to direct effects of allergens on the epithelium c. Predisposition to an altered innate immune sensing that skew responses to type 2 inflammation d. In general, host genetics determine the allergenic activity 6. Patient's gut/lung microbiota

However, the reasons why the IgE-binding frequency (specially that detected *in vitro*) is associated with the clinical impact are not so clear, at least scientifically. Under a commercial perspective, it is important to know which allergen sensitizes most patients because this information can guide the investment on products, although that was probably reasonable before precision medicine. Also, it can be said that the IgE-binding is a crucial parameter for selecting reagents for immunotherapy, but it would make no sense to define the reagents for treating a particular patient based on the frequency of IgE-binding in the population. Therefore, this classification can be confusing for physicians; for example, what should they do if a patient is sensitized to a “minor” allergen? For precision medicine the important is that *the patient* is sensitized, and the allergen has a demonstrated clinical impact. Major and minor allergen are concepts that describe a variable property of allergens (depending on the geographical region and other factors) but not necessarily have clinical importance. Even more, now they can be an obstacle for the development of precision allergology.

## The Rising of Precision Allergology

Precision medicine is the result of a clear historical trend of medical practice, which is to obtain better diagnoses, treatments and preventions. It has been improved by using several tools such as biological markers, including RNA and protein expression, genetic variants and epigenetic marks. These advances are opening doors for predicting outcomes that could be prevented and to identify endotypes susceptible to be treated more accurately and at earlier disease stages, increasing efficacy and reducing costs.

The management of allergic diseases has always been focused on personalized diagnosis and treatment, but there are still many aspects to be resolved. Allergic diseases, including asthma, are ecological problems where the environment selects genetically susceptible individuals that could be affected. The genetic component has not been resolved and the current information is not yet useful for predicting the disease and apply pharmacogenomics criteria (9). Among a wide variety of environmental risk factors, inflammation from exposure to allergens is critical for the inception and triggering of symptoms. In asthma, HDM allergens, cockroaches, molds, pet dander, and some pollens are risk factors, although, as expected, the relative importance of each of these sources depends on the geographic region. In this regard, two important advances have arisen; the use of component resolved diagnosis (CRD) and the characterization of the allergenic activity of several of those components (10).

## Component Resolved Diagnosis, Major and Minor Allergens

CRD allows to accurately define the genuine sensitization to allergens. As sensitizations vary according to the region and other factors (Table 1), there are two options for constructing the platforms for diagnosing allergic diseases: making regional arrays or having arrays that include all the known allergens. In any case, selecting the components based on their IgE-binding frequency (for example, including only major allergens) is contrary to precision medicine, especially to personalized diagnosis and treatment. The selection of components should be guided by the clinical impact of allergens, an aspect related to IgE-binding but not to IgE-binding frequency. It is known that there are allergens with high IgE-binding frequency but low clinical impact, and allergens with low IgE-binding frequency and high clinical impact for some patients (11–14). Since CRD is a necessary step for future component resolved immunotherapy, it is crucial that the arrays include clinically relevant allergens, defined by several parameters in addition to IgE-binding (10).

## Advances in Allergen Characterization

Allergen characterization has several objectives, mainly to evaluate the clinical impact of IgE-binding molecules. By the times the issue of major allergen began, skin tests were the link with allergenic activity, which is the allergen's capacity to induce inflammation. Parallel with the advances in biosciences, allergen characterization has become a more complete, although not yet finished task. The evaluation of the cellular and molecular effects of allergens has revealed that, in addition to the well-known effects of proteases such as Der p 1 (15), Der p 3, and Der p 9 (16) on innate immune pathways, other allergens are able to activate innate inflammation. Of course, these non-IgE mediated mechanisms are not detected by IgE-binding tests, remaining out of any IgE-binding based classification.

Innate immune pathways activation by non-protease allergens has been an important advance in allergen characterization. The first report showing this type of mechanism was the ability of Der p 2 to activate pro-inflammatory signals through TLR-4 in bronchial epithelial cells (17), a report followed by other effects of this allergen (18–22). Among other HDM allergens that can induce non-IgE mediated pro-inflammatory activation of bronchial epithelium are Der p 5 (23), Der p 13 (24), and Blo t 7 (25). More recently, the effects of group 13 allergens on bronchial epithelial cells through activation of serum amyloid A1 protein pathways were reported (8). Important to say that most of these allergens are considered “minor” allergens due to their IgE-binding frequency, although they could be important for susceptible patients, inducing permanent inflammation of bronchial epithelium under appropriate exposure conditions.

A crucial step for understanding the clinical relevance of allergens is to differentiate their allergenicity (the property of inducing and binding IgE antibodies) from their allergenic activity, which means their capacity to induce inflammation, whatever the underlying mechanisms (7, 10). The strength of the IgE response varies between individuals and between allergens (for example HDM vs. food allergens) and reflects the allergenicity; not always correlating with allergenic activity. An update of the research on allergenic activity of indoor allergens was done recently, suggesting that, as occur with the characterization of the allergenic activity of extracts, a set of assays and criteria could help to determine the allergenic activity and clinical impact of IgE-binding molecules (10). Employing these assays, it seemed rational to accept that those IgE antibody binding components with positive *in vivo* provocation tests in humans (which might need GMP-grade batches of allergen), plus positive association in case-control studies and defined proinflammatory mechanisms of action, have a demonstrated allergenic activity, a property close to clinical relevance.

## Discussion

IgE sensitization against allergens is a crucial risk factor for asthma and other allergic diseases. IgE sensitization can lead to clinical symptoms if it occurs in individuals with the susceptible genotype. However, other allergen properties, such as direct effects on the epithelium, also increase the level of inflammation. Sensitization, as well as clinical impact of individual allergens depends on several environmental and genetic factors (Table 1), most of them still undefined for individual molecules. Therefore, what needs to be determined for each individual allergen is its allergenic activity. For a long time, IgE sensitization was used as a proxy of allergenic activity, but now it is known that this is not enough. We propose to stop using the major and minor allergen classification; instead, we should call ***allergens*** those IgE-binding molecules with proven allergenic activity; the rest would simply be called IgE-binding molecules. Allergenic activity would be confirmed if the IgE-binding molecules accomplishes all the following criteria: at least one positive *in vivo* provocation tests in humans, at least one positive *in vitro* provocation test, positive association with an allergic disease in case-control studies and defined proinflammatory mechanisms of action.

Defining the allergenic activity of all IgE-binding molecules from all sources might sound daunting, but the current trends of molecular allergology show that this can be done. Major and minor allergen classification has survived because it was supposed to be useful during a chapter in the history of allergology. It is time to realize that we are living another chapter where the days of major and minor allergens are over. The frequency of IgE-binding, an important epidemiological information of allergens, should be expressed only as a percentage, without labeling it as major or minor.

## Author Contributions

LC wrote the manuscript. RV, NA, and JZ revised, corrected, and approved the manuscript. All authors contributed to the article and approved the submitted version.

## Conflict of Interest

The authors declare that the research was conducted in the absence of any commercial or financial relationships that could be construed as a potential conflict of interest.
